# Genetic variation of *ESR1 *and its co-activator *PPARGC1B *is synergistic in augmenting the risk of estrogen receptor-positive breast cancer

**DOI:** 10.1186/bcr2817

**Published:** 2011-01-26

**Authors:** Yuqing Li, Yi Li, Sara Wedrén, Guoliang Li, Tze Howe Charn, Kartiki Vasant Desai, Carine Bonnard, Kamila Czene, Keith Humphreys, Hatef Darabi, Kristjana Einarsdóttir, Tuomas Heikkinen, Kristiina Aittomäki, Carl Blomqvist, Kee Seng Chia, Heli Nevanlinna, Per Hall, Edison T Liu, Jianjun Liu

**Affiliations:** 1 Human Genetics, Genome Institute of Singapore, 60 Biopolis Street, Singapore 138672, Singapore; 2 Institute for Environmental Medicine, Karolinska Institutet, Box 210, Stockholm 17177, Sweden; 3 Computational & Mathematical Biology, Genome Institute of Singapore, 60 Biopolis Street, Singapore 138672, Singapore; 4 Cancer Biology, Genome Institute of Singapore, 60 Biopolis Street, Singapore 138672, Singapore; 5 Laboratory of Human Embryology, Institute of Medical Biology of Singapore, 8A Biomedical Grove, Singapore 138648, Singapore; 6 Department of Medical Epidemiology and Biostatistics, Karolinska Institutet, Box 281, Stockholm, 17177, Sweden; 7 Centre for Health Services Research, School of Population Health, University of Western Australia, 35 Stirling Highway, Perth, Western Australia 6009, Australia; 8 Departments of Obstetrics and Gynecology, Helsinki University Central Hospital, P.O. Box 700, Helsinki, 00029 HUS, Finland; 9 Clinical Genetics, Helsinki University Central Hospital, P.O. Box 700, Helsinki, 00029 HUS, Finland; 10 Oncology, Helsinki University Central Hospital, P.O. Box 700, Helsinki, 00029 HUS, Finland; 11 Department of Epidemiology and Public Health, Yong Loo Lin School of Medicine, National University of Singapore, MD3, 16 Medical Drive, Singapore 117597, Singapore

## Abstract

**Introduction:**

Given the role of estrogen in breast carcinogenesis and the modification of estrogen receptor (ER) activity by its biochemical cofactors, we hypothesize that genetic variation within ER cofactor genes alters cellular response to estrogen exposure and consequently modifies the risk for ER-positive breast cancer.

**Methods:**

We genotyped 790 tagging SNPs within 60 ER cofactor genes in 1,257 cases and 1,464 controls from Sweden and in 2,215 cases and 1,265 controls from Finland, and tested their associations with either ER-positive or ER-negative breast cancer.

**Results:**

Seven SNPs showed consistent association with ER-positive breast cancer in the two independent samples, and six of them were located within *PPARGC1B*, encoding an ER co-activator, with the strongest association at rs741581 (odds ratio = 1.41, *P *= 4.84 × 10^-5^) that survived Bonferroni correction for multiple testing in the combined ER-positive breast cancer sample (*P*_corrected _= 0.03). Moreover, we also observed significant synergistic interaction (*P*_*interaction *_= 0.008) between the genetic polymorphisms within *PPARGC1B *and *ESR1 *in ER-positive breast cancer. By contrast, no consistent association was observed in ER-negative breast cancer. Furthermore, we found that administration of estrogen in the MCF-7 cell line induced *PPARGC1B *expression and enhanced occupancies of ER and RNA polymerase II within the region of SNP association, suggesting the upregulation of *PPARGC1B *expression by *ESR1 *activation.

**Conclusions:**

Our study revealed that DNA polymorphisms of *PPARGC1B*, coding a *bona fide *ER co-activator, are associated with ER-positive breast cancer risk. The feed-forward transcriptional regulatory loop between *PPARGC1B *and *ESR1 *further augments their protein interaction, which provides a plausible mechanistic explanation for the synergistic genetic interaction between *PPARGC1B *and *ESR1 *in ER-positive breast cancer. Our study also highlights that biochemically and genomically informed candidate gene studies can enhance the discovery of interactive disease susceptibility genes.

## Introduction

It is known that the risk of breast cancer is related to lifetime exposure to estrogen [1,2]. Estrogen stimulates cell proliferation and increases the frequency of spontaneous mutations, leading to a malignant phenotype [3]. Breast cells respond to estrogen via estrogen receptors (ERs) through a defined biochemical process: upon ligand binding, ERs undergo a conformational change that facilitates receptor dimerization, DNA binding, recruitment of ER cofactors, and modulation of target gene expression [4-6].

Endocrine therapy provides strong evidence that attenuation of ER (*ESR1*) activity can reduce breast cancer risk [7], and women with ER-positive tumor would be the most likely to benefit from these treatments [7,8]. The genetic studies of *ESR1*, however, have had contradictory results. Only recently, through a very large genetic association study, has there been demonstrated a small but significant association of polymorphisms within *ESR1 *with the risk of breast cancer [9-11]. Two plausible explanations for the inconsistent results might be due to the small sample sizes and thus limited statistical power of these studies, or that the risk was not evaluated by stratifying breast cancer patients based on tumor ER status. However, there is at least one further possibility: ER cofactors can either enhance transcriptional activity of ER as co-activators or inhibit the activity as co-repressors. The genetic variants within ER cofactors have not been systematically investigated in term of association with breast cancer risk, although some coding variants within individual genes, such as *NCOA3 *and *CCND1*, have been investigated [12-15].

Given the modification of ER activity by its cofactors through their physical and functional interactions [16], the cofactor proteins that bind to ER may be as important as the receptor itself in mediating transcriptional response to estrogen exposure [17]. We therefore hypothesized that genetic variation within ER cofactor genes may alter cellular response to estrogen exposure and consequently, alone or by interacting with genetic variations within *ESR1*, modify breast cancer risk in an ER status-dependent fashion. To assess this hypothesis, we investigated the association of common genetic variation, using a tagging SNP approach, within 60 cofactor genes in two large case-control samples of breast cancer from Sweden and Finland, and investigated their interaction with genetic variation within *ESR1 *in terms of influencing the risk of hormone-driven breast cancer.

## Materials and methods

### Study population

The Swedish sample was from a population-based case-control study that has been described in detail previously [18]. Briefly, 1,322 cases were Swedish-born women diagnosed with incident primary invasive breast cancer between October 1993 and March 1995 who contributed blood samples. All cases were postmenopausal and between 50 and 74 years of age at diagnosis. All the cases were identified through the six regional cancer registries in Sweden. The controls (*n *= 1,524) were randomly selected from the Swedish Registry of Total Population with no previous breast cancer and were frequency-matched for age with the cases. Questionnaires were used to collect risk factor information.

The Finnish sample was from a hospital-based case-control study in which the cases consisted of two series of unselected breast cancer patients and additional familial patients diagnosed at the Helsinki University Central Hospital. The first set of cases were 884 patients collected in 1997/1998 and 2000, covering 79% of all newly diagnosed breast cancer cases during those periods [19,20]. The second set of cases, consisting of 986 newly diagnosed breast cancer patients, were collected during 2001 to 2004 and covered 87% of all such patients during that period [21]. An additional 538 familial breast cancer cases were also collected at the same hospital, as previously described [22,23]. Women with a prior diagnosis of breast cancer *in situ *were excluded, leaving 2,215 invasive breast cancer cases for analysis. Healthy female population controls (*n *= 1,287) were collected from the same geographical regions of Finland as the cases.

Information on reproductive and hormonal risk factors was available for the Swedish sample and showed expected association patterns with breast cancer [24-26]. Such information was not available for the Finnish controls.

Hormone receptor status information was retrieved from medical records of all participating cases and was available for both the Swedish and Finnish cases.

Approval for the study was obtained from the Institutional Review Boards in Sweden, Finland and the National University of Singapore. All subjects provided written informed consent.

### DNA isolation

DNA was extracted from 4 ml whole blood using the QIAamp DNA Blood Maxi Kit (Qiagen, Hilden, Germany) according to the manufacturer's instructions.

### Candidate gene and tagging SNP selection

In the present study, the keywords 'ER cofactor', 'ER coactivator' and 'ER corepressor' were used in a literature search to identify ER cofactor genes. Boolean searching ('AND' 'OR') was used to narrow or broaden the search in PubMed. Using this method, 60 ER cofactor genes were identified as candidate genes. Tagging SNPs within the 60 candidate genes were selected based on the HapMap CEU data (Rel #22/phase II Apr07, on NCBI B36 assembly, dbSNP b126) [27]. In brief, for each gene, all common SNPs with a minor allele frequency >0.05 within the gene and 5 kb surrounding region were first identified from the HapMap database [28]. Tagging SNPs were then selected in Haploview version 4.1 [29] using a pair-wise SNP tagging approach with *r*^2 ^> 0.8 used as the criterion for selection. A total of 806 tagging SNPs were selected within the 60 ER co-factor genes.

### Genotyping

Illumina's GoldenGate assay was used for genotyping SNPs, following the manufacturers' instructions (Illumina, San Diego, CA, USA). In brief, all 806 tagging SNPs were subjected to genotyping assay design, out of which 790 SNPs were successfully designed and subjected to genotyping analysis. DNA samples were randomly assigned to the plates carrying positive and negative controls, and all genotyping results were generated and checked by laboratory staff unaware of the case-control status. SNPs with a call rate <96% (81 SNPs failed in the Swedish sample and 42 SNPs failed in the Finnish sample) and minor allele frequency <1% (18 SNPs in the Swedish sample and 40 SNPs in the Finnish sample) were excluded from further analysis. Deviation of genotype frequencies from those expected under Hardy-Weinberg Equilibrium were assessed in the control subjects. SNPs with Hardy-Weinberg Equilibrium *P *< 7.4 × 10^-5 ^(0.05/675) were excluded (6 SNPs failed in the Swedish sample and 15 SNPs failed in the Finnish sample). In total, 685 SNPs from the Swedish sample and 693 SNPs from the Finnish sample were used for statistical analysis, and 675 shared SNPs between the Swedish and Finnish samples were used for analysis in the combined sample.

Genotyping was duplicated in 2% of samples (in both Swedish and Finnish samples) and there was concordance in >99% of the duplicated samples, suggesting high genotyping accuracy. With *r*^2 ^> 0.8, the average coverage of common variation (minor allele frequency >5%) within the 60 candidate genes was 91%. Out of these, 51 genes had coverage over 80% (Additional file 1 Table S1).

### Reverse transcriptase-quantitative PCR analysis

MCF-7 cells were cultured in DMEM (Invitrogen, Carlsbad, CA, USA) medium with 10% FBS (Invitrogen). Prior to hormone treatment, cells were maintained in phenol-red free DMEM F-12 containing 5% charcoal stripped serum for 72 hours for hormone depletion. Cells were treated with 10 nM 17β-estradiol (Sigma-Aldrich, St. Louis, MO, USA) for a period of 0 or 3 hours. Cells were harvested and total RNA and reverse transcriptase-quantitative PCR analysis was carried out as described previously [30]. Dimethylsulfoxide (Sigma-Aldrich, St. Louis, MO, USA)/vehicle-treated cells were used as controls for the same time course. Real-time PCR analysis was performed in the ABI Prism 7700 sequence detection system using SYBR Green from ABI (Applied Biosystems, Foster City, CA,USA).

Primers were designed using the online Primer 3 program [31]. All experiments were repeated at least twice. Two sets of primers were used for identifying different isoforms of *PPARGC1B*. The oligonucleotide sequences were as follows: PPARGC1B_1 isoform (NM_001172699.1) forward 5'-GAAGAGGAAGAAGGGGAGGA-3' and reverse 5'-CTCTGGTAGGGGCAGTGGT-3'; and PPARGC1B_2 isoform (NM_133263.3) forward 5'-CCTGAAGATGACGTGGGTCT-3' and reverse 5'-CCTTCCTTCTGGGTGTCAGA-3'. β-Actin specific primers (forward 5'-TCCCTGGAGAAGAGCTACGA-3' and reverse 5'-AGGAAGGAAGGCTGGAAGAG-3') were used as an internal control to normalize the amounts of reverse transcribed product used in the PCR reaction. Threshold cycle (Ct) values obtained for *PPARGC1B *isoforms were normalized to β-actin Ct values. The normalized Ct (ΔCt) values were then used to calculate the difference (ΔΔCt) between estradiol-treated and dimethylsulfoxide-treated samples. The fold change of *PPARGC1B *was calculated as 2^-ΔΔCt^.

### Statistical analysis

To measure the magnitude of association between SNPs and breast cancer risk, per-allele odds ratios (ORs) (assuming a log-additive model) and 95% confidence intervals were estimated using logistic regression. As the controls were younger than cases in the Finnish samples, age at diagnosis/enrollment (as a continuous variable) was included in the regression models in the Finnish analysis for OR adjustment. The Cochran-Armitage trend test was used to calculate *P *values in the Swedish and Finnish sample sets, separately in subtypes, and in cases overall. Inverse variance weighting was used in a meta-analysis for two independent datasets. The individual OR was obtained from age-unadjusted analysis in the Swedish sample and age-adjusted analysis in the Finnish sample. To evaluate differences in ORs between studies, a test of homogeneity was carried out for each individual SNP analysis (data not shown).

To determine the model of inheritance, associations between SNPs within the *PPARGC1B *gene and ER-positive breast cancer risk were estimated by assuming dominant, recessive and additive models in the two sample sets. We then performed these analyses with meta-analysis using inverse variance weighting approach. Individual ORs from two independent studies followed-up age-unadjusted analysis in the Swedish sample and age-adjusted analysis in the Finnish sample.

Forward stepwise logistic regression was used to explore whether the associations at the six SNPs were independent of each other. The selection criterion was *P *< 0.2. The analysis was performed in ER-positive breast cancer risk in the two sample sets separately as well as in the combined ER-positive sample dataset. To account for different minor allele frequencies in the two populations, a binary indicator variable for study was included in the regression models as well as age in the combined data regression analysis.

Pair-wise interaction analysis was performed under a dominant mode of inheritance using logistic regression and likelihood ratio tests. To maximize the statistical power, we pooled sample sets from the Swedish and Finnish data. Age and study were included in the model as covariables. The full model included an interaction term between the two interacting variables for the risk of breast cancer. In this multivariate logistic regression analysis, each coefficient provided an estimate of the log OR whilst adjusting for all other variables included in the model. Likelihood ratio tests, comparing models with and without the interaction term, were used to generate *P *values.

All analyses were performed using STATA version 8.0 (StataCorp, College station, TX, USA). Linkage disequilibrium (LD) calculation was performed in Haploview version 4.1 [29]. All statistical tests were two-sided.

## Results

### Study subjects

Two independent case-control samples of breast cancer from Sweden and Finland were investigated in the present study, whose characteristics are summarized in Table 1. The cases and controls of the Swedish sample were frequency-matched on age, whereas the Finnish controls were younger than the Finnish cases (*P *< 0.0001). In the Swedish sample, there were significant differences between the cases and controls in terms of age at first birth (*P *= 0.0002), age at menopause (*P *= 0.0001), hormone replacement treatment use (*P *= 0.017), and parity (*P *= 0.0001), which is consistent with the well-established role of these reproductive factors in breast cancer development. The reproductive factor information was not available for the Finnish controls. In both the Swedish and Finnish cases, there were similar percentages of ER-positive (81.9% vs. 80.9%) and ER-negative (18.1% vs. 19.1%) cases.

**Table 1:** Selected characteristics of cases and controls in the Swedish and the Finnish samples

	Swedish sample			Finnish sample		
		
Characteristic	Number	Mean	*P *value^a^	Number	Mean	*P *value^a^
Entire study						
Age (years)	1,257/1,464	63.11/63.12	0.96	2,214/1,265	56.07/40.88	< 1.00 × 10^-4^
Age at first birth	1,072/1,321	25.50/24.76	2.00 × 10^-4^	1,185/-	26.43/-	/
Age at menopause	1,247/1,460	50.6/49.97	1.00 × 10^-4^	1,341/-	50.34/-	/
BMI (recent)	1,250/1,443	25.71/25.67	0.81	1,525/-	25.03/-	/
	
	**Number**	**Percentage**		**Number**	**Percentage**	
	
Case only						
All cases	1,257	/		2,215	/	
ER-positive	684	81.92		1,709	80.92	
ER-negative	151	18.08		403	19.08	
Controls	1,464	/		1265	/	

Data presented as cases/controls. BMI, body mass index; ER, estrogen receptor. ^a^Two-sided *t *test was used for *P *value estimation.

### SNP association analysis

First, single SNP association analyses were performed using trend tests in the Swedish and Finnish samples separately by stratifying the cases into ER-positive and ER-negative groups, with 685 SNPs being tested in the Swedish sample and 693 SNPs being tested in the Finnish sample. 48 SNPs (7.00%) in the Swedish sample and 50 SNPs (7.28%) in the Finnish sample showed association with ER-positive breast cancer risk with nominal *P *< 0.05. Seven SNPs showed consistent association between the two independent samples (Additional file 1 Table S2), and six of them were located within the *PPARGC1B *gene. In contrast, 21 and 50 SNPs showed association with ER-negative breast cancer with nominal *P *< 0.05 in the Swedish and Finnish samples, respectively, but no SNPs showed consistent associations between the two independent samples.

We then analyzed SNP associations in the combined Swedish and Finnish samples. In general, SNPs showed stronger evidence of association with ER-positive breast cancer than ER-negative breast cancer (Table 2; see also Additional file 1 Table S3). The most significant association was identified at rs741581 within the second intron of *PPARGC1B *(OR = 1.41, *P *= 4.84 × 10^-5^) in ER-positive breast cancer, which survived the Bonferroni correction for multiple testing (*P*_corrected _= 0.03). rs741581 was one of the seven SNPs that showed consistent associations between the Swedish and Finnish samples.

**Table 2:** Twenty-five most significant SNPs associated with ER-positive breast cancer in Swedish and Finnish samples

Gene	SNP	Position	***P *value**^**a**^	**Adjusted *P *value**^**b**^	**OR (95% CI)**^**a**^
*PPARGC1B*	rs741581^cd^	chr5:149182978	4.84 × 10^-5^	0.033	1.414 (1.197, 1.672)
*PPARGC1B*	rs1012543^cd^	chr5:149157138	9.98 × 10^-5^	0.067	1.223 (1.105, 1.353)
*PPARGC1B*	rs6895698^d^	chr5:149120455	2.73 × 10^-4^	0.184	1.225 (1.098, 1.366)
*CARM1*	rs1529711	chr19:10884434	4.26 × 10^-4^	0.288	1.229 (1.096, 1.378)
*RBM23*	rs7469 ^c^	chr14:22440037	1.15 × 10^-3^	0.778	1.248 (1.092, 1.427)
*PPARGC1B*	rs4705365^cd^	chr5:149093146	1.78 × 10^-3^	-	1.193 (1.068, 1.333)
*NCOR2*	rs10846670	chr12:123456184	2.67 × 10^-3^	-	0.872 (0.798, 0.954)
*RBM23*	rs3811187^c^	chr14:22439134	2.96 × 10^-3^	-	1.158 (1.051, 1.275)
*PELP1*	rs4790674	chr17:4529772	3.23 × 10^-3^	-	1.171 (1.054, 1.3)
*PPARGC1B*	rs2340621^d^	chr5:149122509	4.00 × 10^-3^	-	1.149 (1.045, 1.262)
*CCND1*	rs649392^cd^	chr11:69173974	4.43 × 10^-3^	-	0.877 (0.801, 0.96)
*PPARGC1B*	rs10036538^d^	chr5:149135781	5.51 × 10^-3^	-	1.156 (1.043, 1.28)
*PELP1*	rs7214635	chr17:4547769	5.67 × 10^-3^	-	1.166 (1.046, 1.3)
*PPARGC1B*	rs4705382	chr5:149161559	6.32 × 10^-3^	-	0.872 (0.791, 0.962)
*NEDD4*	rs11071224^c^	chr15:53902817	6.32 × 10^-3^	-	0.764 (0.63, 0.927)
*MED13*	rs4968469^c^	chr17:57491867	6.90 × 10^-3^	-	1.145 (1.038, 1.263)
*NCOR2*	rs12321007	chr12:123449054	7.44 × 10^-3^	-	1.137 (1.035, 1.25)
*MED13*	rs9889324^c^	chr17:57481404	8.89 × 10^-3^	-	1.14 (1.033, 1.258)
*NCOR2*	rs1794973	chr12:123391545	9.45 × 10^-3^	-	0.889 (0.813, 0.972)
*PPARGC1B*	rs1422429	chr5:149146627	1.11 × 10^-2^	-	1.124 (1.027, 1.231)
*NCOR2*	rs10846666	chr12:123450306	1.17 × 10^-2^	-	0.867 (0.776, 0.969)
*NCOA1*	rs17046513	chr2:24817999	1.26 × 10^-2^	-	1.284 (1.055, 1.563)
*NCOA1*	rs17046462	chr2:24759054	1.37 × 10^-2^	-	1.285 (1.053, 1.568)
*NCOR2*	rs10846667	chr12:123450377	1.40 × 10^-2^	-	0.895 (0.819, 0.978)
*SNW1*	rs3759728^c^	chr14:77299912	1.49 × 10^-2^	-	0.855 (0.753, 0.97)

ER, estrogen receptor; chr, chromosome; OR, odds ratio; CI, confidence interval. ^a^*P *value and OR were obtained from meta-analysis based on the inverse variance method for two independent datasets. The individual OR was obtained from age-unadjusted analysis in the Swedish sample and age-adjusted analysis in the Finnish sample. ^b^*P *value was adjusted by Bonferroni correction (*n *= 675); -, adjusted *P *> 1. ^c^SNP belongs to the top 25 most significant SNPs associated with ER-negative breast cancer. ^d^SNP was significantly associated with ER-positive breast cancer in both the Swedish and Finnish datasets.

We also evaluated the SNP association with overall breast cancer risk and found 55 SNPs (8.03%) from the Swedish samples and 61 SNPs (8.80%) from the Finnish samples to show association with overall breast cancer risk with nominal P < 0.05. Only two SNPs, however, showed consistent association between the two independent samples (Additional file 1 Table S2), and none of the associations survived Bonferroni correction for multiple testing in the combined samples (smallest *P*_corrected _= 0.198).

### Genotype association analysis of *PPARGC1B *in ER-positive breast cancer

*PPARGC1B *is located on 5q33.1 and encodes for peroxisome proliferative activated receptor gamma coactivator beta protein (PGC-1β), a *bona fide *co-activator of ERα. In the present study, 40 tagging SNPs within *PPARGC1B *were successfully genotyped in both the Swedish and Finnish samples, which could capture 80% of common variants (131 out of 162 SNPs) within *PPARGC1B *with a minimal *r*^2 ^value of 0.8 (mean *r*^2 ^value = 0.95, according to HapMap CEU data).

To have a better understanding of the association within *PPARGC1B*, we performed genotype-based association analysis by assuming dominant, recessive and additive model of inheritance. We found that the top three SNPs yielding the most significant association evidence in the dominant model compared with other models (Additional file 1 Table S5). Under the dominant model, the same six SNPs (as for the trend tests) of the 40 SNPs within *PPARGC1B *showed consistent association with ER-positive breast cancer between the Swedish and Finnish samples (Table 3). The strength of the association (ORs) at the six SNPs was stronger in ER-positive breast cancer than in overall or ER-negative cancers, with the strongest association identified at rs741581 (*P *= 1.9 × 10^-2 ^in the Swedish samples, *P *= 6.1 × 10^-5 ^in the Finnish samples, and *P *= 1.8 × 10^-5 ^in the combined samples).

**Table 3:** Six overlapping SNPs in *PPARGC1B *associated with ER-positive breast cancer in Swedish and Finnish samples

SNP	Allele^a^	Subtype	Swedish sample		Finnish sample	
				
			MAF^b^	OR^c ^(95% CI)	MAF^b^	OR^c ^(95% CI)
rs4705365	G/A	ER+	0.21	1.26 (1.05, 1.52)	0.17	1.26 (1.05, 1.52)
		ER-		1.14 (0.81, 1.61)		1.17 (0.9, 1.52)
		All cases		1.14 (0.97, 1.33)		1.18 (0.99, 1.4)
rs6895698	G/A	ER+	0.22	1.27 (1.06, 1.53)	0.17	1.39 (1.15, 1.67)
		ER-		1.05 (0.74, 1.48)		1.14 (0.88, 1.49)
		All cases		1.12 (0.96, 1.3)		1.25 (1.05, 1.49)
rs2340621	G/A	ER+	0.31	1.3 (1.08, 1.57)	0.32	1.22 (1.03, 1.46)
		ER-		0.86 (0.61, 1.2)		1.05 (0.82, 1.34)
		All cases		1.12 (0.96, 1.31)		1.14 (0.97, 1.34)
rs10036538	C/G	ER+	0.26	1.19 (0.99, 1.42)	0.22	1.2 (1, 1.43)
		ER-		0.91 (0.65, 1.28)		1.05 (0.81, 1.35)
		All cases		1.03 (0.89, 1.2)		1.11 (0.94, 1.31)
rs1012543	A/G	ER+	0.26	1.26 (1.05, 1.51)	0.23	1.26 (1.06, 1.5)
		ER-		1.08 (0.77, 1.51)		1.08 (0.84, 1.38)
		All cases		1.11 (0.95, 1.29)		1.18 (1, 1.39)
rs741581	G/A	ER+	0.08	1.32 (1.05, 1.67)	0.05	1.76 (1.33, 2.31)
		ER-		0.81 (0.49, 1.32)		1.21 (0.81, 1.82)
		All cases		1.12 (0.92, 1.37)		1.53 (1.18, 1.98)

ER+, estrogen receptor-positive; ER-, estrogen receptor-negative; MAF, minor allele frequency; OR, odds ratio; CI, confidence interval. ^a^Major allele/minor allele. ^b^From control samples only. ^c^ORs were performed on a dominant model, age-unadjusted analysis in the Swedish sample and age-adjusted analysis in the Finnish sample.

**Figure 1:**
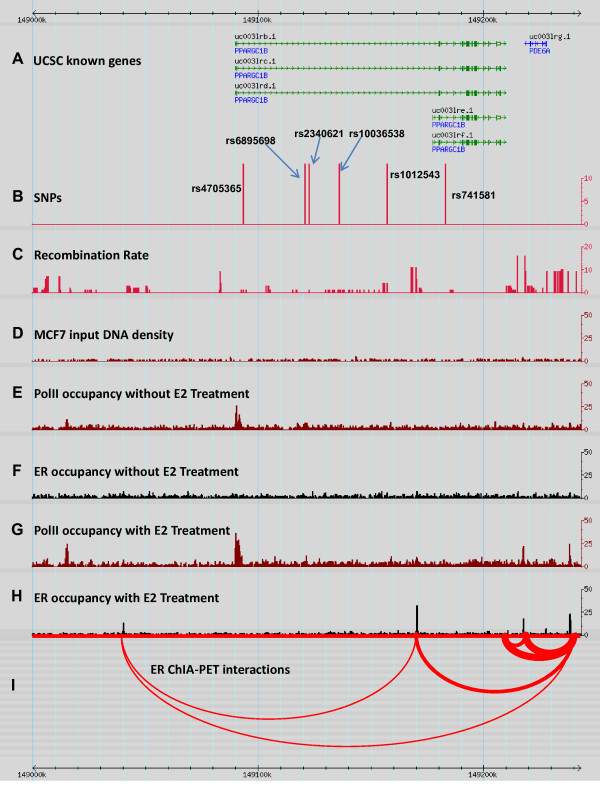
**Transcriptional regulation of *PPARGC1B *by estrogen receptor alpha in the MCF7 cell line**. **(A) **Schematic diagram of the genes from the UCSC database. **(B) **Map positions of six significant SNPs within *PPARGC1B*. **(C) **Recombination rate surrounding *PPARGC1B *from the HapMap CEU database. **(D) **MCF7 input DNA density for chromatin immunoprecipitation-sequencing (CHIP-seq) analysis. **(E) **CHIP-seq RNA *Pol*II occupancy density without 17β-estradiol (E2) treatment. **(F) **CHIP-seq estrogen receptor (ER) occupancy without E2 treatment. **(G) **CHIP-seq RNA *Pol*II occupancy density with E2 treatment. **(H) **CHIP-seq ER occupancy with E2 treatment. **(I) **ER interaction loop detected by chromatin interaction analysis by paired-end tag sequencing (ChIA-PET).

The six SNPs showing consistent association with ER-positive breast cancer were located within two regions of high LD (Figure 1B), suggesting that the associations at those SNPs may not be completely independent. We therefore performed a forward stepwise logistic regression (cut-off *P *= 0.20) and revealed two independent associations with ER-positive breast cancer at rs741581 (*P *= 0.031) and rs6895698 (*P *= 0.014) in the combined sample. Under the dominant model, we found that rs741581, rs6895698, age and study sample were four independent variables associated with ER-positive breast cancer risk. Similarly, the same stepwise analysis of ER-positive breast cancer in the two individual samples also revealed two independent associations at rs741581 (*P *= 0.172) and rs2340621 (*P *= 0.036) in the Swedish sample and at rs741581 (*P *= 0.023) and rs6895698 (*P *= 0.053) in the Finnish sample. Notably, rs6895698 and rs2340621 lie within the same LD block and are highly correlated (*r*^2 ^= 0.72, according to HapMap CEU data).

### Association of *ESR1 *variation with ER-positive breast cancer

Our previous study suggested an association between *ESR1 *polymorphisms and breast cancer risk [9,32] in the same Swedish sample. The association was within a region flanked by rs988328 to rs3020318 and was manifested by three haplotypes. Using the haplotype information from the HapMap CEU data, we identified three common SNPs that were in high LD (*r*^2 ^= 0.89) with one of the three haplotypes (TAG18~21) [9], while no SNPs were found with *r*^2 ^> 0.5 for the other two haplotypes, based on the HapMap CEU data (Rel #22/phase II Apr07, on NCBI B36 assembly, dbSNP b126). Given that the three SNPs were in perfect LD (*r*^2 ^= 1), we genotyped one of the three SNPs, rs7761846, in our Swedish and Finnish samples. Given that a large association study of *ESR1 *by the Breast Cancer Association Consortium also revealed a significant association within the same region under a dominant model [10], we searched for SNPs that were in high LD (*r*^2 ^> 0.5) with the three haplotypes but were not genotyped in our previous study. Then we performed a genotype-based association analysis under a dominant model of inheritance. As expected, rs7761846 showed association with ER-positive breast cancer (OR = 1.28, *P *= 0.014) in the combined sample. The two independent Swedish and Finnish samples also revealed consistent association, although the association in the Finnish sample did not reach statistical significance (Table 4).

**Table 4:** Association analysis of rs7761846 within *ESR1 *under a dominant model in ER-positive case analysis

Sample	Control	Case	OR (95% CI)	*P *value
Swedish	1,442	675	1.43 (1.10, 1.86)	0.007
Finnish	1,246	1669	1.10 (0.81, 1.48)	0.55
Combined^a^	2,688	2344	1.28 (1.05, 1.56)	0.014

ER, estrogen receptor; OR, odds ratio; CI, confidence interval. ^a^Combined analysis was performed on logistic regression adjusted by age and study.

### Genetic interaction between the polymorphisms of *PPARGC1B *(rs6895698, rs2340621 and rs741581) and *ESR1 *(rs7761846)

Given the known modification of ER activity by *PPARGC1B *in cellular response to estrogen exposure, we investigated the genetic interaction between rs741581, rs2340621 and rs6895698 within *PPARGC1B *and rs7761846 within *ESR1 *in terms of modulating ER-positive breast cancer risk. The analysis in the combined sample identified a significant synergistic interaction between rs2340621 (representing *PPARGC1B*) and rs7761846 (representing *ESR1*) (*P*_interaction _= 0.008) (Table 5). Women carrying both *PPARGC1B *(rs2340621) and *ESR1 *(rs7761846) risk genotypes (*GA/AA *and *CT/CC*) had a much higher risk for breast cancer than noncarriers (*GG *and *TT*) (OR = 1.94, *P *= 2.03 × 10^-6^). Similar patterns of genetic interaction were also observed between the remaining SNPs rs741581 (*PPARGC1B*) and rs7761846 (*ESR1*) as well as rs6895698 (*PPARGC1B*) and rs7761846 (*ESR1*), although these interactions did not achieve statistical significance - probably due to the low minor allele frequencies of rs741581 and rs6895698. However, the significant genetic interactions could not be detected in overall or ER-negative breast cancer (Additional file 2, Tables S6 and S7).

**Table 5:** Pair-wise interaction between SNPs within *PPARGC1B *and *ESR1 *on ER-positive breast cancer in combined Swedish and Finnish samples

*ESR1 *(rs7761846)	*PPARGC1B*					
	
	Cases (%)	Controls (%)	OR (95% CI)	Cases (%)	Controls (%)	OR (95% CI)
	GG (rs2340621)			GA/AA (rs2340621)		
		
TT	916 (39)	1,121 (42)	1	1,160 (50)	1,280 (48)	1.18 (1.03, 1.34)
CT/CC	106 (5)	151 (6)	0.95 (0.71, 1.29)	161 (7)	134 (5)	1.94 (1.47, 2.55)
Interaction *P *value^a^	0.008					
		
	GG (rs6895698)			GA/AA (rs6895698)		
		
TT	1,284 (55)	1,566 (58)	1	791 (34)	837 (31)	1.28 (1.11, 1.47)
CT/CC	160 (7)	184 (7)	1.21 (0.93, 1.55)	106 (5)	101 (4)	1.77 (1.30, 2.42)
Interaction *P *value^a^	0.506					
		
	GG (rs741581)			GA/AA (rs741581)		
		
TT	1,741 (74)	2,076 (77)	1	335 (14)	326 (12)	1.41 (1.17, 1.70)
CT/CC	225 (10)	251 (9)	1.25 (1.01, 1.55)	42 (2)	34 (1)	2.18 (1.32, 3.59)
Interaction *P *value^a^	0.459					

OR, odds ratio; CI, confidence interval. ^a^Analysis was performed on combined dataset, in which study and age were regarded as covariables.

### Transcriptional regulation of *PPARGC1B *by ERα

To understand the molecular mechanism underlying the observed genetic interaction, we investigated whether there was any transcriptional cross-talk between the two genes beyond the known ligand-dependent, co-activating interaction of the PGC-1β with ERα [33,34], using the ER-responsive MCF7 breast cancer cell line.

First, we examined the expression of *PPARGC1B *in MCF7 and noted a twofold induction of *PPARGC1B *expression by ER activation after estradiol administration (Additional file 3 Figure S1). As a marker of transcriptional activity, chromatin immunoprecipitation-sequencing analysis in the same MCF7 cell line identified a significant peak of RNA polymerase II occupancy close to the transcriptional start site of *PPARGC1B *within the LD region of SNP association, and the RNA polymerase II occupancy was further enhanced by estradiol treatment. This observation confirms the transcriptional responsiveness of *PPARGC1B *to estradiol. Moreover, the chromatin immunoprecipitation-sequencing analysis also identified five ER binding sites in and around *PPARGC1B *(one site located approximately 50 kb 5' of the transcriptional start site, one in the second intron of the gene within 13 kb of the associated SNP rs741581, and the other three binding sites approximately 10, 31 and 57 kb 3' of the polyadenylation signal sequence) and within the LD region of significant association with ER-positive breast cancer (Figure 1F). Interestingly, the sites showing highest of ER occupancy were seen at two locations, one ~13 kb from the significant SNP rs741581 and the second within 31 kb 3' of the polyadenylation signal sequence.

We recently described the identification of all ER binding site interactions in the human genome [35,36] and defined that genes engaged in chromatin loop formation by a transcription factor were definitively regulated by the factor. Our data indicated that all of the ER binding sites around *PPARGC1B *were engaged in chromatin loop formation centered on the *PPARGC1B *gene (Figure 1I), which indicates that ERα directly regulates *PPARGC1B*.

Taken together, these data strongly indicate that *PPARGC1B *expression could be directly regulated by ERα and - when coupled with the known enhancement of ERα transcriptional activity by the PGC-1β at the site of binding - suggest a feed-forward regulatory loop between the two genes that augments ER signaling when the two factors are present.

## Discussion

To our knowledge, this is the first comprehensive association analysis of common variation within ER cofactor genes in breast cancer where 36 ER co-activators and 24 ER co-repressors were investigated. The utilization of two independent case-control samples of northern European origin allowed us to identify an association based not only on the overall significance in the large combined sample, but also on the consistency of the SNP association between the two individual samples. We found significant associations between *PPARGC1B *polymorphisms and risk for ER-positive breast cancer, and, importantly, we revealed a synergistic effect between the genetic polymorphisms within *PPARGC1B *and *ESR1*.

Genetic association studies of ER cofactor genes have so far been limited. Burwinkel and colleagues reported a significant association of coding variants Q586 H and T960T of *NCOA3 *with familial breast cancer risk, and further suggested that familial breast cancer patients may condense the rare allele's contribution to the protective effect of breast cancer [12]. Whilst two studies have reported an association of the variant Pro241Pro in *CCND1 *with breast cancer risk [37,38], other studies have reported negative results for this variant [14,39,40]. In particular, Wirtenberger and colleagues investigated the coding variant Ala203Pro of *PPARGC1B *and found it to be associated with familial breast cancer susceptibility [41]. In our study, we did not observe significant association between polymorphisms in *NCOA3 *and *CCND1 *with breast cancer risk. The Ala203Pro (rs7732671) variant of *PPARGC1B*, however, is 10 kb away and not correlated with *PPARGC1B *SNP rs741581 (*r*^2 ^< 0.05 in HapMap CEU data), and thus would not have been detected by our tagging SNP approach. Nevertheless, both Wirtenberger and colleagues' study and our study support the association of genetic variation of *PPARGC1B *with particular subtypes of breast cancer.

Importantly, the association of *PPARGC1B *as well as its synergistic interaction with *ESR1 *was only observed in breast cancer patients with ER-positive tumors, as would be expected according to the biochemical mechanism of interaction. There is growing evidence that the impact of genetic risk factors on breast cancer varies by hormone receptor status. For example, recent studies by the Breast Cancer Association Consortium have led to the discovery of novel breast cancer susceptibility loci in *FGFR2*, *TNRC9*, 8q24, 2q35, and 5p12 that showed stronger association with ER-positive disease than with ER-negative disease [42-45], with fibroblast growth factor receptor also being a direct target of ER. These data suggest the risk of ER-positive tumors that has been shown to be driven by reproductive factors in epidemiologic studies also has a genomic basis based on the constituents of the ER gene regulatory network [46,47]. In our study, although the sample sizes of two ER-positive datasets were smaller compared with the two overall datasets, the number of overlapping SNPs between the Swedish and Finnish studies was thus larger than that observed in the overall breast cancer analysis. Recently, we also demonstrated that genetic variation of the estrogen metabolism pathway - particularly the genes involved in the production of estrogen through androgen conversion - also influences the risk for the development of estrogen-sensitive breast cancer [48]. As with this study, the effect size of the metabolism gene polymorphisms are relatively small but, taken together with *PPARGC1B *and fibroblast growth factor receptor, show that the estrogen receptor signaling axis that engages both upstream and downstream components may have, in the composite, a significant role in the genesis of the most common form of breast cancer.

The genetic interaction between *PPARGC1B *and *ESR1 *is biologically plausible. The *PPARGC1B *protein PGC-1β is a *bona fide *ER co-activator [34] that physically interacts with ERα and plays a role in amplifying ER signaling, which provides a convincing biological mechanism for the observed genetic interaction between the two genes. Furthermore, our series of transcriptional regulation analyses in the MCF7 ER-positive breast cancer cell line has demonstrated that *PPARGC1B *expression can be induced by estrogen treatment, and this transcriptional response of *PPARGC1B *is probably mediated by five functional ER binding sites around *PPARGC1B *that are all engaged in interlocking chromatin loops highly indicative of an ER regulated gene [35]. *PPARGC1B *may thus be involved in a feed-forward control mechanism with ERα such that ER induction (for example, by estradiol treatment) heightens the expression of a co-activator *PPARGC1B *of ER, which in turn increases ER action at the DNA binding site. The feed-forward looping mechanism will therefore further augment the protein interaction between *PPARGC1B *and *ESR1*. This putative amplification effect, if confirmed, is another mechanistic model for epistatic interactions between genetic loci and may be one reason for the strength of its signal in the association study as compared with the other ER cofactors studied.

There are some limitations to our study. Coverage of common variation is not sufficient (< 80%) for some genes (Additional file 1, Table S1), so that some associations may have been missed. In addition, our tagging SNP selection provides a rather limited coverage of 5 kb surrounding sequences of the candidate genes, which may have contributed to some associations of regulatory SNPs being undetected, such as the one reported within *ESR1 *[11]. The number of overlapping SNPs between the two datasets is small for both ER-positive and overall breast cancer analyses. The limited overlapping could be due to ethnic heterogeneity between the two population samples and their moderate sample sizes. On the one hand, the ethnic heterogeneity may partially explain the low overlapping SNPs between two datasets; on the other hand, the current sample size is not large enough to capture the moderate effect of associated SNPs. Some of the top SNPs for each individual sample set are therefore probably false positive, which causes the small overlap between the numbers of significant SNPs in both datasets. The sample size limitation in ER-negative patients also could lead to the nonsignificant results in ER-negative analysis, since we observed that some associations in ER-negative analysis are in the same direction with ER-positive analysis. ER cofactors are known to work as a multicomponent protein complex, but due to a sample size limitation we are unable to detect interaction among three or more genes simultaneously. It is also worth noting that the contribution of genetic variation to cancer risk is based on both their prevalence and penetrance, and thus the relative importance of individual SNPs may vary from population to population. Further confirmation of our findings in other populations is therefore warranted.

## Conclusions

Our study has revealed an association of genetic variation within *PPARGC1B *with the risk of ER-positive breast cancer. Consistent with the known interaction of *PPARGC1B *and ER at the molecular level, where *PPARGC1B *modulates ER activity and thus ER signaling, our study revealed a synergistic effect between genetic variation within the *PPARGC1B *and *ESR1 *genes. *PPARGC1B *has been shown to alter responses to the selective ER modulator, tamoxifen [33]. Kressler and colleagues also demonstrated that *PPARGC1B *indirectly co-activates tamoxifen-bound ERα, which cooperates with *NCOA1 *to enable tamoxifen agonism in kidney and osteosarcoma cell lines. Lastly, the synergism demonstrated in the present study also suggests that disrupting the interaction between an ER co-activator - such as *PPARGC1B *- and ERα, or blocking their mutual activation, may represent a sensitive and leveraged strategy for cancer prevention [7]. Our study therefore provides new biological insight into the genetic basis of the more common ER-positive breast cancer and highlights that biochemically and genomically informed candidate gene study can enhance the discovery of interactive disease susceptibility genes.

## Abbreviations

*CCND1*: cyclin D_1_; Ct: threshold cycle; DMEM: Dulbecco's modified Eagle's medium; ER: estrogen receptor; *ESR1*: estrogen receptor 1; FBS: fetal bovine serum; *FGFR2*: fibroblast growth factor receptor 2; *NCOA*: nuclear receptor coactivator; OR: odds ratio; PCR: polymerase chain reaction; PGC-1β: peroxisome proliferative activated receptor gamma coactivator beta protein; *PPARGC1B*: peroxisome proliferative activated receptor gamma coactivator beta; SNP: single nucleotide polymorphism; *TNRC9*: TOX high mobility group box family member 3.

## Competing interests

The authors declare that they have no competing interests.

## Authors' contributions

YQL, SW, KE, HN, KSC, PH, ETL and JJL initiated and designed the study. CB, KC and HN provided the study material and patient information. YQL, CB, TH, KA and SW collected and organized the data. YQL, YL, GLL, THC, DKV, JJL, KH and HD performed data analysis and interpreted results. YQL, SW, YL, DKV, KH, PH, ETL and JJL drafted the manuscript. CB, HD, KH, KE, HN, PH, ETL and JJL performed critical review and revised the manuscript. All authors read and approved the manuscript.

## Supplementary Material

Additional file 1 **Supplementary results of coverage evaluation of common variants and association analysis in ER cofactor genes**. Table S1 presenting coverage evaluation of the common variant in 60 ER cofactor genes. Table S2 presenting ORs and *P *values of the consistent SNPs between the Swedish and Finnish samples from the analyses of ER-positive and overall breast cancer. Table S3 presenting the 25 most significant SNPs in ER-negative association analysis in Swedish and Finnish samples. Table S4 presenting the 25 most significant SNPs in overall association analysis in Swedish and Finnish samples. Table S5 presenting the comparison of *P *value among additive, dominant and recessive models in the analysis of ER-positive breast cancer in *PPARGC1B *in the combined Swedish and Finnish samples. Click here for file

Additional file 2 **Analysis of the pair-wise interaction effect between SNPs within *PPARGC1B *and *ESR1 *on the overall and ER-negative breast cancer in the combined Swedish and Finnish samples**. Table S6 presenting analysis of the pair-wise interaction effect between SNPs within *PPARGC1B *and *ESR1 *on the overall breast cancer in the combined Swedish and Finnish samples. Table S7 presenting the analysis of pair-wise interaction effect between SNPs within *PPARGC1B *and *ESR1 *on the ER-negative breast cancer in the combined Swedish and Finnish samples. Click here for file

Additional file 3 **Relative expression of PPARGC1B gene in MCF7 cells 3 hrs post E2 treatment**. Figure S1 presenting a relative expression of the *PPARGC1B *gene in MCF7 cells 3 hours post 17β-estradiol treatment. Click here for file
