# Supplementary material for: Genetic variation of ESR1 and its co-activator PPARGC1B is synergistic in augmenting the risk of estrogen receptor-positive breast cancer
Source: Breast Cancer Res. 2011 Jan 26;13(1):R10. doi: 10.1186/bcr2817 (PMC3109578; doi:10.1186/bcr2817)
Supplement: Additional file 1 — Supplementary results of coverage evaluation of common variants and association analysis in ER cofactor genes. Table S1 presenting coverage evaluation of the common variant in 60 ER cofactor genes. Table S2 presenting ORs and P values of the consistent SNPs between the Swedish and Finnish samples from the analyses of ER-positive and overall breast cancer. Table S3 presenting the 25 most significant SNPs in ER-negative association analysis in Swedish and Finnish samples. Table S4 presenting the 25 most significant SNPs in overall association analysis in Swedish and Finnish samples. Table S5 presenting the comparison of P value among additive, dominant and recessive models in the analysis of ER-positive breast cancer in PPARGC1B in the combined Swedish and Finnish samples. [file bcr2817-S1.DOC]

**Table S1**.Coverage evaluation of common variant in 60 ER cofactor genes

| **Gene** | **#Tag** | **Successful genotyped tags #** | **Captured SNPs # (r^2 >0.8)** | **Total SNPs #** | **Coverage^*^** | **Mean max r^2 ^†^** |
| --- | --- | --- | --- | --- | --- | --- |
| ARA70 | 6 | 5 | 10 | 11 | 90% | 0.993 |
| Calmodulin1 | 7 | 4 | 7 | 14 | 50% | 0.995 |
| Calmodulin2 | 8 | 6 | 20 | 24 | 83% | 0.968 |
| Calmodulin3 | 6 | 4 | 4 | 6 | 66% | 1 |
| UBE3A | 7 | 7 | 48 | 48 | 100% | 0.977 |
| NCOA2 | 24 | 27 | 154 | 154 | 100% | 0.985 |
| RPL7 | 5 | 5 | 7 | 7 | 100% | 0.985 |
| SNW1 | 11 | 8 | 42 | 48 | 87% | 0.985 |
| BAG1 | 3 | 2 | 7 | 10 | 70% | 0.985 |
| SUPT6H | 4 | 4 | 7 | 7 | 100% | 0.985 |
| SMARCE1 | 3 | 3 | 18 | 18 | 100% | 0.985 |
| TRIM24 | 9 | 8 | 47 | 48 | 97% | 0.985 |
| KAT5 | 54 | 47 | 229 | 248 | 92% | 0.985 |
| MED13 | 8 | 6 | 29 | 31 | 93% | 0.985 |
| TRIP4 | 7 | 7 | 25 | 25 | 100% | 0.985 |
| NCOA6 | 5 | 5 | 49 | 49 | 100% | 0.985 |
| CARM1 | 6 | 4 | 13 | 16 | 81% | 0.985 |
| PRMT1 | 4 | 3 | 4 | 5 | 80% | 0.985 |
| CALCOCO1 | 10 | 8 | 26 | 28 | 92% | 0.985 |
| NEDD4 | 31 | 34 | 128 | 128 | 100% | 0.985 |
| PPARGC1B | 51 | 40 | 131 | 162 | 80% | 0.985 |
| RBM39 | 5 | 4 | 36 | 39 | 92% | 0.985 |
| RBM23 | 8 | 7 | 20 | 21 | 95% | 0.985 |
| RBM14 | 3 | 2 | 2 | 3 | 66% | 0.985 |
| CCND1 | 5 | 4 | 5 | 6 | 83% | 0.985 |
| CEBPB | 5 | 4 | 3 | 3 | 100% | 0.985 |
| CREBBP | 21 | 18 | 23 | 23 | 100% | 0.985 |
| NCOA1 | 23 | 19 | 106 | 106 | 100% | 0.985 |
| NCOA7 | 38 | 33 | 115 | 115 | 100% | 0.985 |
| PPARG | 35 | 30 | 112 | 112 | 100% | 0.985 |
| DDX5 | 7 | 5 | 16 | 16 | 100% | 0.985 |
| EP300 | 10 | 10 | 42 | 42 | 100% | 0.985 |
| NCOA3 | 20 | 18 | 98 | 98 | 100% | 0.985 |
| PELP1 | 9 | 6 | 18 | 18 | 100% | 0.985 |
| PPARGC1A | 55 | 46 | 97 | 100 | 97% | 0.985 |
| SRA1 | 6 | 5 | 11 | 11 | 100% | 0.985 |
| NCoR1 | 4 | 3 | 57 | 74 | 77% | 0.985 |
| HDAC7 | 14 | 10 | 12 | 19 | 63% | 0.985 |
| SHARPIN | 3 | 3 | 6 | 6 | 100% | 0.985 |

**Table S1.** Coverage evaluation of common variant in 60 ER cofactor genes

(Continued)

| **Gene** | **#Tag** | **Successful genotyped tags #** | **Captured SNPs # (r^2 >0.8)** | **Total SNPs #** | **Coverage^*^** | **Mean max r^2 ^†^** |
| --- | --- | --- | --- | --- | --- | --- |
| SAFB | 5 | 4 | 14 | 18 | 77% | 0.966 |
| SAFB2 | 10 | 9 | 15 | 16 | 93% | 0.985 |
| NRIP1 | 9 | 8 | 17 | 20 | 85% | 0.967 |
| LCOR | 5 | 5 | 38 | 38 | 100% | 0.962 |
| COUP-TF | 3 | 3 | 4 | 4 | 100% | 1 |
| DDX54 | 3 | 2 | 7 | 8 | 87% | 1 |
| NSD1 | 10 | 8 | 38 | 43 | 88% | 0.977 |
| MTA1 | 4 | 4 | 5 | 5 | 100% | 0.991 |
| PHB2 | 3 | 2 | 1 | 1 | 100% | 1 |
| FOXO1 | 11 | 9 | 32 | 34 | 94% | 0.962 |
| NR2C1 | 13 | 11 | 23 | 27 | 85% | 0.985 |
| GMPR2 | 5 | 4 | 6 | 8 | 75% | 1 |
| TAF1B | 18 | 15 | 136 | 148 | 91% | 0.959 |
| SMAD4 | 3 | 3 | 24 | 24 | 100% | 0.993 |
| SIAH2 | 4 | 2 | 4 | 6 | 66% | 0.957 |
| ESR2 | 20 | 20 | 70 | 70 | 100% | 0.966 |
| BRCA1 | 7 | 5 | 42 | 46 | 91% | 0.991 |
| NCOR2 | 93 | 67 | 131 | 144 | 90% | 0.972 |
| NROB2 | 7 | 5 | 6 | 6 | 100% | 1 |
| RBFOX2 | 21 | 16 | 21 | 21 | 100% | 0.982 |
| NR0B1 | 12 | 9 | 9 | 9 | 100% | 0.993 |
| Total | 806 | 675 |  |  |  |  |

#Tag, number of tagging SNP; SNP: single nucleotide polymorphism; r^2 , the square of the correlation coefficient

Criteria: MAF>=0.05

* : Number of captured SNPs divide total SNPs number

† : Average r^2 of those captured SNPs

T**able S2.** ORs and P values of the consistent SNPs between the Swedish and Finnish samples from the analyses of ER+ and overall breast cancer.

| Subtype | Gene | SNP | Position | OR* (95%CI)_Swedish | P value^†^ Swedish | OR* (95%CI)_Finnish | P value^†^ Finnish |
| --- | --- | --- | --- | --- | --- | --- | --- |
| ER+ | PPARGC1B | rs741581 | chr5:149182978 | 1.266 (1.019,1.573) | 0.034 | 1.662 (1.279,2.16) | 4.06E-5 |
|  | PPARGC1B | rs6895698 | chr5:149120455 | 1.183 (1.017,1.375) | 0.032 | 1.273 (1.087,1.492) | 0.002 |
|  | PPARGC1B | rs1012543 | chr5:149157138 | 1.215 (1.054,1.401) | 0.008 | 1.231 (1.066,1.422) | 0.005 |
|  | PPARGC1B | rs4705365 | chr5:149093146 | 1.196 (1.026,1.394) | 0.024 | 1.190 (1.014,1.397) | 0.029 |
|  | PPARGC1B | rs2340621 | chr5:149122509 | 1.203 (1.05,1.378) | 0.008 | 1.100 (0.965,1.254) | 0.037 |
|  | CCND1 | rs649392 | chr11:69173974 | 0.873 (0.766,0.995) | 0.042 | 0.881 (0.779,0.998) | 0.043 |
|  | PPARGC1B | rs10036538 | chr5:149135781 | 1.158 (1.004,1.336) | 0.048 | 1.153 (0.996,1.334) | 0.048 |
| overall | CARM1 | rs1529711 | chr19:10884434 | 1.178 (1.02,1.362) | 0.026 | 1.225 (1.062,1.413) | 0.006 |
|  | RBM23 | rs7469 | chr14:22440037 | 1.213 (1.013,1.451) | 0.037 | 1.273 (1.086,1.493) | 0.006 |

SNP: single nucleotide polymorphism rs id ;chr: chromosome; OR, odds ratio; 95% CI, 95% confidence interval; ER+, estrogen receptor positive

*: Odds ratio and corresponding 95% confidence interval which is allele-based test. OR from individual study was calculated based on age-unadjusted in Swedish and age-adjusted in Finnish.

^†^: P-value of association using CA trend-test (rounded to 3 decimals)

**Table S3.** Twenty-five most significant SNPs in ER negative association analysis in Swedish and Finnish samples

| **Gene** | **SNP** | **Position** | **P-value**^†^ | **Adjusted P-value**^‡^ | **OR^†^(95% CI)** |
| --- | --- | --- | --- | --- | --- |
| RBM23 | rs3811187* | chr14:22439134 | 5.800E-04 | 0.392 | 1.217 (1.088,1.361) |
| RBM23 | rs7469* | chr14:22440037 | 1.120E-03 | 0.756 | 1.299 (1.11,1.52) |
| MED13 | rs16945724 | chr17:57372670 | 1.180E-03 | 0.797 | 1.292 (1.107,1.508) |
| NEDD4 | rs11071224* | chr15:53902817 | 0.0024 | – | 0.682 (0.533,0.873) |
| NEDD4 | rs16976653 | chr15:54001512 | 0.002 | – | 0.695 (0.555,0.87) |
| MED13 | rs4968469* | chr17:57491867 | 0.002 | – | 1.203 (1.073,1.348) |
| CCND1 | rs649392* | chr11:69173974 | 0.002 | – | 0.846 (0.762,0.94) |
| RBM23 | rs8012963 | chr14:22435114 | 0.002 | – | 0.829 (0.735,0.935) |
| MED13 | rs9889324* | chr17:57481404 | 0.002 | – | 1.194 (1.065,1.339) |
| HDAC7 | rs2544030 | chr12:46481718 | 0.006 | – | 0.847 (0.753,0.952) |
| CCND1 | rs11603541 | chr11:69181554 | 0.007 | – | 0.822 (0.712,0.948) |
| NEDD4 | rs12915953 | chr15:54062835 | 0.007 | – | 0.714 (0.559,0.913) |
| HDAC7 | rs2525051 | chr12:46482206 | 0.008 | – | 0.869 (0.784,0.964) |
| CALCOCO1 | rs3741658 | chr12:52396000 | 0.009 | – | 0.859 (0.766,0.963) |
| PPARGC1B | rs1012543* | chr5:149157138 | 0.011 | – | 1.164 (1.035,1.308) |
| CCND1 | rs603965 | chr11:69172091 | 0.014 | – | 1.138 (1.027,1.261) |
| NR2C1 | rs7297570 | chr12:93995461 | 0.014 | – | 0.849 (0.746,0.968) |
| NR2C1 | rs12316136 | chr12:93996220 | 0.015 | – | 1.164 (1.03,1.316) |
| NCOA7 | rs1322754 | chr6:126195857 | 0.016 | – | 1.247 (1.042,1.493) |
| ESR2 | rs17225885 | chr14:63764502 | 0.018 | – | 0.711 (0.536,0.944) |
| NR2C1 | rs249144 | chr12:93970755 | 0.018 | – | 1.175 (1.028,1.344) |
| PPARGC1B | rs4705365* | chr5:149093146 | 0.018 | – | 1.166 (1.027,1.325) |
| PPARGC1B | rs741581* | chr5:149182978 | 0.018 | – | 1.255 (1.039,1.516) |
| NCOA7 | rs9482710 | chr6:126229812 | 0.018 | – | 1.239 (1.038,1.479) |
| SNW1 | rs3759728* | chr14:77299912 | 0.022 | – | 0.843 (0.729,0.976) |

chr: chromosome; SNP: single nucleotide polymorphism rs id; OR, odds ratio; 95% CI, 95% confidence interval

^*^:SNP belongs to top 25 most significant SNPs associated with ER positive breast cancer.

^†^:P-value and odds ratio were obtained from meta-analysis based on inverse variance method. Individual OR was obtained from age-unadjusted in Swedish and age-adjusted in Finnish.

^‡^:P-value was adjusted by Bonferroni correction (n=675); "–", adjusted P-value >1

**Table S4**: Twenty-five most significant SNPs in overall association analysis in Swedish and Finnish samples.

| **Gene** | **SNP** | **Position** | **P-value^†^** | **Adjusted P-value^‡^** | **OR^†^(95% CI)** |
| --- | --- | --- | --- | --- | --- |
| RBM23 | rs7469 | chr14:22440037 | 2.930E-04 | 0.198 | 1.246 (1.106,1.404) |
| CARM1 | rs1529711 | chr19:10884434 | 4.039E-04 | 0.273 | 1.202 (1.085,1.33) |
| RBM23 | rs3811187 | chr14:22439134 | 0.002 | – | 1.145 (1.051,1.247) |
| PELP1 | rs7214635 | chr17:4547769 | 0.005 | – | 1.148 (1.043,1.262) |
| CCND1 | rs649392 | chr11:69173974 | 0.005 | – | 0.892 (0.825,0.966) |
| ESR2 | rs17225885 | chr14:63764502 | 0.006 | – | 0.745 (0.604,0.917) |
| NR2C1 | rs11107870 | chr12:93958583 | 0.006 | – | 0.832 (0.73,0.949) |
| NCOA3 | rs450110 | chr20:45724889 | 0.006 | – | 0.81 (0.696,0.942) |
| NCOR2 | rs10846670 | chr12:123456184 | 0.006 | – | 0.897 (0.83,0.97) |
| MED13 | rs4968469 | chr17:57491867 | 0.007 | – | 1.127 (1.034,1.23) |
| MED13 | rs9889324 | chr17:57481404 | 0.008 | – | 1.125 (1.031,1.227) |
| NR2C1 | rs12316136 | chr12:93996220 | 0.010 | – | 1.132 (1.031,1.244) |
| PELP1 | rs4790674 | chr17:4529772 | 0.010 | – | 1.129 (1.03,1.238) |
| PPARGC1B | rs741581 | chr5:149182978 | 0.012 | – | 1.212 (1.044,1.407) |
| SNW1 | rs3759728 | chr14:77299912 | 0.013 | – | 0.871 (0.781,0.971) |
| ESR2 | rs1255998 | chr14:63763624 | 0.013 | – | 0.85 (0.748,0.966) |
| MED13 | rs16945801 | chr17:57471169 | 0.014 | – | 1.129 (1.024,1.244) |
| NCOR2 | rs12321007 | chr12:123449054 | 0.015 | – | 1.109 (1.02,1.205) |
| NCOR2 | rs10846667 | chr12:123450377 | 0.016 | – | 0.908 (0.84,0.982) |
| PPARGC1B | rs1012543 | chr5:149157138 | 0.017 | – | 1.115 (1.019,1.219) |
| PPARGC1A | rs7656250 | chr4:23475114 | 0.020 | – | 1.123 (1.018,1.238) |
| ESR2 | rs8018687 | chr14:63763835 | 0.020 | – | 0.798 (0.659,0.965) |
| PPARGC1A | rs2970869 | chr4:23502467 | 0.021 | – | 0.896 (0.816,0.983) |
| PPARGC1A | rs1388332 | chr4:23438412 | 0.023 | – | 1.162 (1.021,1.323) |
| NR2C1 | rs249144 | chr12:93970755 | 0.023 | – | 1.127 (1.017,1.248) |

SNP: single nucleotide polymorphism rs id; chr: chromosome; OR, odds ratio; 95% CI; 95% confidence interval

^†^:P-value and odds ratio were obtained from meta-analysis based on inverse variance method. Individual OR was obtained from age-unadjusted in Swedish and age-adjusted in Finnish.

^‡^:P-value was adjusted by Bonferroni correction (n=675); "–", adjusted P-value >1

**Table S5**. Comparison of P value among additive, dominant and recessive models in the analysis of ER positive breast cancer in PPARGC1B in combined Swedish and Finnish samples

| **SNP** | **Position** | **Genotype^*^** | **MAF^†^** | **MAF^‡^** | **Additive model** | **Dominant model** | **Recessive model** |
| --- | --- | --- | --- | --- | --- | --- | --- |
| rs741581 | chr5:149182978 | G/A | 0.089 | 0.062 | 3.45E-05 | 1.18E-05 | 9.08E-01 |
| rs6895698 | chr5:149120455 | G/A | 0.222 | 0.182 | 2.03E-04 | 2.11E-05 | 7.13E-01 |
| rs2340621 | chr5:149122509 | G/A | 0.321 | 0.322 | 3.38E-03 | 3.34E-04 | 6.26E-01 |
| rs1012543 | chr5:149157138 | A/G | 0.268 | 0.241 | 7.29E-05 | 3.88E-04 | 7.14E-03 |
| rs4705365 | chr5:149093146 | G/A | 0.215 | 0.176 | 1.41E-03 | 5.61E-04 | 4.49E-01 |
| rs10036538 | chr5:149135781 | C/G | 0.263 | 0.229 | 4.44E-03 | 6.37E-03 | 1.11E-01 |
| rs17653473 | chr5:149163569 | C/A | 0.312 | 0.309 | 1.31E-02 | 1.45E-02 | 1.86E-01 |
| rs4705382 | chr5:149161559 | G/A | 0.317 | 0.285 | 5.25E-03 | 1.54E-02 | 4.02E-02 |
| rs1422429 | chr5:149146627 | G/A | 0.414 | 0.378 | 1.25E-02 | 1.82E-02 | 9.81E-02 |
| rs11738917 | chr5:149135232 | G/C | 0.057 | 0.057 | 9.62E-02 | 4.71E-02 | – |
| rs4705367 | chr5:149105188 | G/A | 0.109 | 0.108 | 8.43E-02 | 9.99E-02 | 5.76E-01 |
| rs4705377 | chr5:149143607 | G/A | 0.473 | 0.493 | 1.07E-01 | 1.48E-01 | 2.36E-01 |
| rs12516673 | chr5:149085798 | A/T | 0.234 | 0.256 | 4.96E-01 | 1.69E-01 | 2.55E-01 |
| rs13174179 | chr5:149130864 | G/A | 0.295 | 0.322 | 2.19E-01 | 1.74E-01 | 6.86E-01 |
| rs26120 | chr5:149212406 | G/A | 0.184 | 0.234 | 2.33E-01 | 2.58E-01 | 5.02E-01 |
| rs17797713 | chr5:149095348 | A/G | 0.217 | 0.192 | 1.50E-01 | 2.65E-01 | 1.56E-01 |
| rs1076064 | chr5:149092359 | A/G | 0.338 | 0.375 | 3.61E-01 | 3.01E-01 | 7.46E-01 |
| rs26123 | chr5:149211525 | C/A | 0.29 | 0.338 | 4.25E-01 | 3.19E-01 | 9.30E-01 |
| rs1107344 | chr5:149211979 | G/A | 0.075 | 0.083 | 3.61E-01 | 3.20E-01 | 9.25E-01 |
| rs17711430 | chr5:149126755 | G/A | 0.034 | 0.027 | 2.93E-01 | 3.23E-01 | – |
| rs1469042 | chr5:149137507 | G/A | 0.427 | 0.426 | 3.93E-01 | 3.73E-01 | 6.37E-01 |
| rs17110439 | chr5:149167650 | G/A | 0.207 | 0.261 | 2.76E-01 | 3.87E-01 | 3.27E-01 |
| rs32587 | chr5:149180796 | A/G | 0.37 | 0.384 | 9.93E-01 | 3.90E-01 | 2.35E-01 |
| rs251464 | chr5:149176427 | G/C | 0.311 | 0.34 | 7.72E-01 | 4.02E-01 | 4.66E-01 |
| rs17653642 | chr5:149201358 | G/A | 0.034 | 0.016 | 3.69E-01 | 4.82E-01 | – |
| rs10875551 | chr5:149153327 | G/A | 0.267 | 0.213 | 2.26E-01 | 4.99E-01 | 7.05E-02 |
| rs10783180 | chr5:149197180 | A/G | 0.105 | 0.099 | 4.10E-01 | 5.17E-01 | 3.63E-01 |
| rs1544744 | chr5:149167231 | G/A | 0.162 | 0.145 | 2.73E-01 | 5.62E-01 | 4.65E-02 |
| rs6888451 | chr5:149104127 | G/C | 0.107 | 0.109 | 4.59E-01 | 5.62E-01 | 4.58E-01 |
| rs17711418 | chr5:149126002 | A/T | 0.084 | 0.13 | 8.25E-01 | 5.70E-01 | 1.99E-01 |
| rs2161257 | chr5:149170190 | A/G | 0.45 | 0.436 | 5.42E-01 | 5.97E-01 | 9.02E-02 |
| rs4705380 | chr5:149155145 | G/A | 0.111 | 0.108 | 4.57E-01 | 6.18E-01 | 3.23E-01 |
| rs9986116 | chr5:149093289 | C/A | 0.226 | 0.258 | 9.57E-01 | 7.17E-01 | 5.30E-01 |
| rs11749217 | chr5:149126515 | C/A | 0.039 | 0.04 | 6.68E-01 | 7.98E-01 | – |
| rs7729592 | chr5:149179849 | G/A | 0.083 | 0.054 | 5.46E-01 | 8.10E-01 | 5.91E-02 |
| rs7725304 | chr5:149203397 | C/A | 0.134 | 0.15 | 7.97E-01 | 8.38E-01 | 8.05E-01 |
| rs2010994 | chr5:149171845 | G/A | 0.267 | 0.243 | 5.52E-01 | 8.75E-01 | 2.43E-01 |
| rs10875552 | chr5:149169682 | A/G | 0.378 | 0.354 | 8.94E-01 | 8.82E-01 | 6.28E-01 |
| rs741580 | chr5:149181863 | A/G | 0.059 | 0.042 | 5.63E-01 | 9.15E-01 | 7.30E-03 |
| rs10515638 | chr5:149132724 | C/A | 0.093 | 0.143 | 8.44E-01 | 9.62E-01 | 3.27E-01 |

ER positive, estrogen receptor positive; SNP: single nucleotide polymorphism rs id; chr: chromosome; MAF, minor allele frequency; OR, odds ratio; 95% CI, 95% confidence interval

*:Genotype: major allele / minor allele.

^†^:MAF in Swedish control

^‡^:MAF in Finnish control
