# Supplementary material for: Genetic variation of ESR1 and its co-activator PPARGC1B is synergistic in augmenting the risk of estrogen receptor-positive breast cancer
Source: Breast Cancer Res. 2011 Jan 26;13(1):R10. doi: 10.1186/bcr2817 (PMC3109578; doi:10.1186/bcr2817)
Supplement: Additional file 2 — Analysis of the pair-wise interaction effect between SNPs within PPARGC1B and ESR1 on the overall and ER-negative breast cancer in the combined Swedish and Finnish samples. Table S6 presenting analysis of the pair-wise interaction effect between SNPs within PPARGC1B and ESR1 on the overall breast cancer in the combined Swedish and Finnish samples. Table S7 presenting the analysis of pair-wise interaction effect between SNPs within PPARGC1B and ESR1 on the ER-negative breast cancer in the combined Swedish and Finnish samples. [file bcr2817-S2.DOC]

**Table S6**: Analysis of pair-wise interaction effect between SNPs within PPARGC1B and ESR1 on the overall breast cancer in the combined Swedish and Finnish samples

| **ESR1 (rs7761846)** | **PPARGC1B** | | | | | |
| --- | --- | --- | --- | --- | --- | --- |
| Genotype | GG (rs2340621) | | | GA/AA (rs2340621) | | |
|  | case (%) | ctrl (%) | OR(95%CI) | case(%) | ctrl(%) | OR(95%CI) |
| TT | 1371 (40) | 1121 (42) | 1 | 1643 (48) | 1280 (48) | 1.08 (0.96, 1.22) |
| CT/CC | 176 (5) | 151 (6) | 1.07 (0.83, 1.38) | 218 (6) | 134 (5) | 1.57 (1.23, 2.01) |
| Interaction P value | 0.087 | | | | | |
|  | GG (rs6895698) | | | GA/AA (rs6895698) | | |
|  | case (%) | ctrl (%) | OR(95%CI) | case(%) | ctrl(%) | OR(95%CI) |
| TT | 1907 (56) | 1566 (58) | 1 | 1106 (32) | 837 (31) | 1.15 (1.02, 1.30) |
| CT/CC | 245 (7) | 184 (7) | 1.23 (0.99, 1.53) | 148 (4) | 101 (4) | 1.45 (1.10, 1.92) |
| Interaction P value | 0.88 | | | | | |
|  | GG (rs741581) | | | GA/AA (rs741581) | | |
|  | case (%) | ctrl (%) | OR(95%CI) | case(%) | ctrl(%) | OR(95%CI) |
| TT | 2564 (75) | 2076 (77) | 1 | 449 (13) | 326 (12) | 1.2 (1.02, 1.42) |
| CT/CC | 337 (10) | 251 (9) | 1.23 (1.02, 1.48) | 57 (2) | 34 (1) | 1.72 (1.09, 2.70) |
| Interaction P value | 0.561 | | | | | |

SNP: single nucleotide polymorphism rs id; OR, odds ratio; 95% CI, 95% confidence interval

Analysis was performed on combined data set, in which study and age were regarded as covariables.

**Table S7:** Analysis of pair-wise interaction effect between SNPs within PPARGC1B and ESR1 on the ER negative breast cancer in the combined Swedish and Finnish samples.

| **ESR1 (rs7761846)** | **PPARGC1B** | | | | | | |
| --- | --- | --- | --- | --- | --- | --- | --- |
| Genotype | GG (rs2340621) | | | | GA/AA (rs2340621) | | |
|  | case (%) | ctrl (%) | OR(95%CI) | | case(%) | ctrl(%) | OR(95%CI) |
| TT | 234 (43) | 1121 (42) | 1 | | 246 (45) | 1280 (48) | 0.95 (0.77, 1.17) |
| CT/CC | 35 (6) | 151 (6) | 1.26 (0.83, 1.92) | | 32 (6) | 134 (5) | 1.39 (0.90, 2.14) |
| Interaction P value | 0.627 | | | | | | |
|  | GG (rs6895698) | | | GA/AA (rs6895698) | | | |
|  | case (%) | ctrl (%) | OR(95%CI) | case(%) | | ctrl(%) | OR(95%CI) |
| TT | 321 (59) | 1566 (58) | 1 | 158 (29) | | 837 (31) | 1.06 (0.85, 1.32) |
| CT/CC | 44 (8) | 184 (7) | 1.34 (0.92, 1.94) | 23 (4) | | 101 (4) | 1.48 (0.90, 2.43) |
| Interaction P value | 0.889 | | | | | | |
|  | GG (rs741581) | | | GA/AA (rs741581) | | | |
|  | case (%) | ctrl (%) | OR(95%CI) | case(%) | | ctrl(%) | OR(95%CI) |
| TT | 426 (78) | 2076 (77) | 1 | 53 (10) | | 326 (12) | 0.94 (0.68, 1.30) |
| CT/CC | 60 (11) | 251 (9) | 1.31 (0.95, 1.81) | 7 (1) | | 34 (1) | 1.72 (0.74, 4.02) |
| Interaction P value | 0.499 | | | | | | |

SNP: single nucleotide polymorphism rs id ; ER negative, estrogen receptor negative; OR, odds ratio; 95% CI, 95% confidence interval

Analysis was performed on combined data set, in which study and age were regarded as covariables.
