# Supplementary material for: Genetic variation of ESR1 and its co-activator PPARGC1B is synergistic in augmenting the risk of estrogen receptor-positive breast cancer
Source: Breast Cancer Res. 2011 Jan 26;13(1):R10. doi: 10.1186/bcr2817 (PMC3109578; doi:10.1186/bcr2817)
Supplement: Additional file 3 — Relative expression of PPARGC1B gene in MCF7 cells 3 hrs post E2 treatment. Figure S1 presenting a relative expression of the PPARGC1B gene in MCF7 cells 3 hours post 17β-estradiol treatment. [file bcr2817-S3.DOC]

**Figure S1:** Relative expression of PPARGC1B gene in MCF7 cells 3 hrs post E2 treatment
